# Some Points in the Pathology and Therapeutics of Digestion

**Published:** 1903-03-07

**Authors:** 


### Hospital Clinics and Medical Progress.

### SOME POINTS IN THE PATHOLOGY AND THERAPEUTICS OF DIGESTION.

In the experimental investigation of digestion by Pawlow and his pupils, important observations were made on the effects of powerful irritants on the mucous membrane of the stomach. Alcohol, corrosive sublimate, silver nitrate and oil of mustard were introduced into the stomach direct in separate experiments. In each case the irritant provoked a copious secretion of mucus from the superficial epithelial cells of the stomach.

The question might be asked, was this an example of acute catarrh of the gastric mucous membrane? If so a definite pathological change was caused by the irritant, and the mucous secretion would persist for some time at least. But the experiments showed that the actual result of the introduction of one of the above-mentioned powerful irritants is *not* to set up a definite condition of acute catarrh. The mucous secretion is intense and immediate. But it does not continue, and may have disappeared by the next day. This secretion is, indeed, the attempt of the superficial structures to ward off and dilute the effect of the irritant. The flow of mucus is distinctly a protective secretion keeping the irritant from attacking the underlying structures. Such a secretion may be compared with the salivary secretion evoked by the presence of acid or fine sand in the mouth. Further it was noted that during the secretion of this mucous fluid the peptic glands are at rest.

The effect of irritants may now be compared with that of a meal of flesh on the secretory apparatus of the stomach. In the former case a purely mucous secretion is evoked, while in the latter a peptic secretion without any excess of mucus is poured out. The two processes are thus separate and distinct.

But the continued application of 10 per cent. $AgNO_3$ solution will produce a condition of asthenia of the peptic glands. When stimulated to secrete by the presence of food the peptic glands now respond by a rapid but comparatively brief secretion. During the first three hours the secretion per hour in each hour is above the normal in amount, but it soon ceases, and the total secretion only reaches to two-thirds of the normal amount. Apparently the gland cells in this condition, in this state of asthenia, are more excitable, but at the same time are more easily and quickly fatigued. They are in an unstable position rapidly responding to stimulation, and quickly becoming used up.

Intense cold has also notable effects on the gland cells of the stomach. It causes a change in the cells resulting in a condition the reverse of that produced by the continued application of 10 per cent. $AgNO_3$ solution. The gland cells are now less easily excitable, and less easily fatigued; and the total secretion evoked by a test meal is one-third as large again, *i.e.*, 38 c.cm. compared with 27 c.cm. There is a difficulty in exciting the glands after prolonged and intense cold, and this corresponds closely with the reversal of the electromotive phenomena of secretion noticed by Biedermann as the effect of cold. The outgoing current of secretory activity being replaced by an ingoing current, from the free border to the basement membrane of the cell.

But one of the most important experiments carried out under Pawlow's direction was the investigation of gastric secretion in a dog suffering from a round ulcer of the stomach.

The gastric ulcer was accompanied by a hypersecretion of gastric juice. This overproduction by the gastric cells amounted to three or four times the normal amount of secretion for a given test meal of bread. Also there was a deviation from the normal rate of secretion. The first hour after the ingestion of food the secretion was approximately normal in amount, but the subsequent secretion was much greater than usual. Indeed the cellular activity of the first hour was maintained in subsequent hours up to the fifth, instead of decreasing after the first hour as is usually the case with a bread diet. This may be an instance of an augmented excitability of the secretory mechanism under pathological conditions.

The point of clinical importance is that this hypersecretion is most marked with a bread diet, pointing to the undesirability of starchy foods in a case of round ulcer of the stomach.

In other cases Pawlow found that the *first* effect of disease was to produce an inhibition of gland-activity, probably by a reflex action. This was followed by a compensatory activity in other parts and other glands. He would, therefore, offer this fact as an explanation of the increased activity of the remainder of the stomach in the case of gastric ulcer. Again, it was found, that when the stomach was prevented from secreting, a compensatory activity of the pancreas carried on the necessary digestion of the food.

The treatment of conditions of hypersecretion of gastric juice by the administration of alkalies was investigated experimentally. The alkalies cause an altered condition of the gland cells themselves. The asthenic excitability is permanently altered, by the alkalies inhibiting their secretory activity, to a considerable degree. Hence in irritable debility of the cell the use of alkalies is singularly appropriate. The clinical use of alkalies in disordered digestion is thus experimentally proved to be founded on physiological facts.

A greater difficulty, however, was experienced in treating conditions of abnormal glandular activity, where hyposecretion was the marked feature, where there was enfeebled activity of the glands.

The introduction of water into the stomach provoked a certain degree of activity, and was distinctly useful in promoting a flow of gastric juice. Here is the explanation of the many good results obtained by the clinical use of lavage of the stomach. Alcohol, too, was found to excite the enfeebled cells to further secretion.

The results obtained by these experiments of Pawlow and his pupils show in no obscure or uncertain manner the immense value of the experimental method. Centuries of observation have accumulated certain empirical facts, but experimental work has been necessary to prove an adequate explanation of their meaning. There is now reason to rejoice with Pawlow that "modern medicine has passed the stage of gruesome experiment on man himself."

